# Approaching the socialist factory and its workforce: considerations from fieldwork in (former) Yugoslavia

**DOI:** 10.1080/0023656X.2017.1244331

**Published:** 2016-10-18

**Authors:** Rory Archer, Goran Musić

**Affiliations:** ^a^ Centre for Southeast European Studies, University of Graz, Graz, Austria

**Keywords:** Yugoslavia, Yugoslav working class, socialism, workers’ self-management, factory sources

## Abstract

The socialist factory, as the ‘incubator’ of the new socialist (wo)man, is a productive entry point for the study of socialist modernization and its contradictions. By outlining some theoretical and methodological insights gathered through field-research in factories in former Yugoslavia, we seek to connect the state of labour history in the Balkans to recent breakthroughs made by labour historians of other socialist countries. The first part of this article sketches some of the specificities of the Yugoslav self-managed factory and its heterogeneous workforce. It presents the ambiguous relationship between workers and the factory and demonstrates the variety of life trajectories for workers in Yugoslav state-socialism (from model communists to alienated workers). The second part engages with the available sources for conducting research inside and outside the factory advocating an approach which combines factory and local archives, print media and oral history.

## Introduction

The socialist factory, not only as the site of employment and production, but also as an important institution of political activity, daily routines and leisure practices, is a fruitful entry point for the exploration of multifaceted aspects of socialist modernization, its contradictions and demise. Envisioned as the main transporter of socialist values, the urban proletariat occupied a privileged position in the ideologies of the ruling communist parties of east, central and south-east Europe, while heavy industry attracted the lion’s share of state investment. The industrial workplace, with its disciplining work rhythms and spread of common responsibilities, was seen as the incubator of the new socialist (wo)man and their progressive consciousness.^1^ The vulgarized understandings of Marxist theory, carrying strong ‘productivist’ and ‘workerist’ undertones, were often imposed upon academia by the party-state. Researchers today, however, are in the position to explore these formerly canonized sites of state socialism with fresh theoretical assumptions. One might approach socialist societies from the institutions the party-states so revered, and the social layer standing so close to its core ideology, in order to debunk the fossilized images of the working class and its workplaces while recognizing that these past projections still weigh heavily on understandings of labour.

This article aims to provide new impetus for the study of working class history in the postsocialist Balkan region by mapping out potentially productive ways to engage with factory sources. As such, it aims to contribute to Sabine Rutar’s appeal for a new history of labour of southeastern Europe, in tune with the latest theoretical insights of global labour history.^2^ We present a number of theoretical and methodological considerations when approaching field research which are derived from an ongoing research project ‘Between class and nation: Working class communities in 1980s Serbia and Montenegro’.^3^ Starting from a micro-level approach (detailed case studies of four factories and their surrounding communities) this project seeks insight into how workers conceived of macro-level processes (the dynamic changes at the level of the state and society) and their role within these. The empirical conclusions of the research project obtained up to this point allow us to sketch some emerging insights regarding the study of the Yugoslav factory and its workforce.

We detail important specificities of the self-managed industrial enterprise and reveal some of the ambivalences present among its blue-collar workforce. The first part of the article stresses the heterogeneity of the working class in spite of the socialist state’s reification of the industrial workforce in official discourse. Following this discussion, we take stock of the array of sources available when attempting to reconstruct dynamics inside the socialist factory. Keeping in mind censorship and party control, questions inevitably arise regarding the accuracy and reliability of meeting proceedings, party reports and factory newspapers produced under state-socialism. What are productive ways to approach and interpret these sources? What are some of the difficulties the researcher encounters when trying to access the sources for socialist enterprises and how might such difficulties be mitigated?

As our research project is focused on Yugoslavia, most of the conclusions are reached based upon the experience of the self-managed factory.^4^ Nonetheless, we consider that the findings can have a broader relevance for researchers studying labour under state socialism. The exceptionality of ‘Yugoslav road to socialism’ is often taken for granted and we believe the micro-historical research along the suggested lines might put this common wisdom to the test by uncovering differences and similarities between the making of urban proletariat in Yugoslavia and in other socialist states. In any case, the task facing researchers of labour in south-east Europe is not simply one of ‘catching up’ with the current state of the art in East-Central Europe, but creatively contributing to a common body of knowledge about varieties of labour practices and working-class subjectivities in state socialism.

Across socialist states the sheer scope of material investment in industry and the political focus placed on the urban proletariat makes it very likely that most of the basic mechanisms of the party-state rule, as well as the main challenges faced by the communists, were reflected inside the factories. This was the logic followed by Ulf Brunnbauer, Visar Nonaj and Biljana Raeva when undertaking comparative research of two steel factories in Albania and Bulgaria. They consider that:

…[s]alient patterns of economic and social organization, but also of cultural and societal policies of the communist regimes are reflected in the relations at the workplace and between workers and the management in these two steel mills. The two enterprises were sites of the construction of communism. They stand for its ideology, but also for the divergent social results that came out of policy measures.^5^

Since the late nineteenth century, the paternalism of factory owners and the spread of corporatist understanding of industrial relations in many states, particularly in central and northern Europe,^6^ encouraged workers’ stronger attachment to the privately owned factory. In spite of such tendencies, the capitalist factory principally remained a place of production. Wageworkers in capitalism normally exited the work premises at the end of the workday with the aim of using part of their leisure time in the market, exchanging wages for material goods and services. Contrary to this, in state socialism the distinction between the factory as a place of production and site of consumption and sociability was frequently more blurred. In societies that upheld work as the key tenet of all political and social rights, and praised manual industrial labour as the principle producer of all social wealth, the factory was an important centre for the organization of everyday life in working-class communities. The workplace was deeply embedded in the welfare regimes of state socialism as the provider of goods and social services guaranteed by the state. Additionally, the industrial plant served as a site of political agitation and a cultural hub. As Dietrich Beyrau points out in the case of Soviet Union, the term ‘work collective’ evoked strong ties between the workers of a single plant where the institution of employment played the role of the ‘educator, controller and the point of emotional attachment’.^7^

In the immediate post-1989 period, social scientists in former socialist countries were wary of tackling the topic of industrial workers and social class. Labour was seen as an obsolete social layer dependent on the totalitarian state, thus helping the preservation and might of the communist rule^8^ and the intellectual climate ‘was profoundly hostile to the writing of working class history’.^9^ Yet, in many ways, it was the revival of research focused on the relationship between the socialist state and industrial labour, which has decisively contributed to altering the view of state socialism as a static system ruled over by monolithic parties.^10^ Indeed, the methodological consideration of the micro politics and negotiation of state power between workers, management and party helped bring about increased sensitivity to the dynamism, nuances and ambiguities of communist labour policy, planned economies and socialist societies in general.

Numerous studies of everyday life in socialist industrial plants and working-class neighbourhoods revealed the concrete limits of the scope of power the socialist regimes could exercise in practice. The theoretical and methodological insights of the cultural turn, *Alltagsgeschichte,* as well as the revitalization of the best traditions of social and labour history, were initially put to use mostly in studies dealing with the German Democratic Republic, but the trend eventually spread among researchers of other state socialist countries.^11^ These scholars were of the view that the general mechanisms of rule and relations between the party-state and the working class are best unwrapped at the local level and approached through the seemingly mundane practices of the actual workforces. Hence, a focus has been made on issues like housing policy, consumption, education, gender, sociability and community building. The micro historical approach enabled these studies to overcome the inherited essentialist presentations of the working class as a politically united and combative historical actor embedded with pre-given purpose. At the same time, such efforts revived the sense of ‘real life’ agency by placing the spotlight on the often subtle and innovative ways in which the workers claimed their rights and negotiated with authorities.

In terms of the periods of socialist rule under scrutiny, the bulk of the social-historical research on labour in central and eastern Europe^12^ has focused on the early post Second World War years and the often coercive attempts to create a new socialist working man and woman through reconstruction, rapid industrialization and the nationalization of existing industry. Alternatively, the period of stirrings in industrial relations, facilitated by the process of destalinization in the second half of 1950s also attracted interest.^13^ In recent years, the steady declassification and ordering of archival holdings finally enables researchers to look into the last three decades of state socialism in more depth. Late socialism, conceived of as the period between destalinization and the terminal crisis of state socialist regimes in the late 1980s,^14^ presents itself as especially suitable for bottom-up research agendas.

The spread of consumerism and the loosening grip of the party-state over public life produced ambiguous results. The material concessions to the working class and the creation of ‘welfare dictatorships’^15^ encouraged quiescence on the shop floor and a tendency towards individualization and withdrawal into the private sphere. This ‘social contract’ depended heavily on the increased availability of consumer goods and leisure opportunities and according to Paulina Bren and Mary Neuburger, it ‘…came to define the 1970s and 1980s’.^16^ However, the increasing influence of the market and growing inequalities between the manual workers on the one hand, and the political and economic elites on the other, made the working class feel increasingly alienated and cynical towards socialist ideology. The makeup of the industrial workforce became more complex during late socialism due to more sophisticated production techniques and extensive education. The rise of knowledge-based hierarchies inside the factories triggered frictions between the manual workers and the technocratic-managerial elites.^17^

Peter Heumos’ overview of the post-1989 studies on industrial labour in former socialist countries shows there is a gap between the state of research in Eastern-Central Europe compared to the Balkan states of Romania and Bulgaria, where researchers have been slower to adopt the trend of socio-historical approaches to studying labour under socialism. Heumos observes that the proletariat in southeastern Europe has a weaker pre-socialist legacy in comparison to its central and eastern Europe counterpart due to late industrialization.^18^ Largely created anew by through socialist economic policies after the Second World War, it is difficult to separate the urban working class in the Balkans from the communist regimes which instigated rapid industrialization and urbanization and thus to accommodate the research of labour with the predominant postsocialist historiographical leitmotivs of national identity and resistance to communist rule. In the case of former Yugoslavia, the historiography of labour has been further undermined by the legacy of war in the 1990s. Often approached as ‘post-conflict’ rather than ‘post-socialist’,^19^ the historiography of late socialism in Yugoslavia has been biased towards elite actors, nationalism and claims of victimhood, with little concern for class-based initiatives and grass-root agency.^20^ The next section will highlight the heterogeneity of the Yugoslav workforce and the divergence of workers’ life courses in late socialism which were kept out of sight by sanctified images of labour under socialism and remain neglected in more recent historiography.

## The peasant, the self-manager and everyone in between

In the Yugoslav brand of socialism, the official identification of the state bureaucracy as the main adversary of working-class interests coupled with the radical decentralization of political and economic life, promoted the autonomous, self-managed enterprise as the nucleus of workers’ daily activities and personal identification. A workplace in the social sector was often the place of primary on-site health care, nutrition (eating meals at the canteen and acquiring foodstuffs through the trade union), political participation (in the institutions of self-management at the level of the enterprise) and means of taking holidays and engaging in leisure pursuits (through the workplace holiday camps, exchanges with other factories and in various clubs for sports and hobbies). The market competition between companies, workers’ participation in the decision-making process and the development of a system of broader political representation structured around the workplace led Susan Woodward to describe the self-managed factory as the ‘centre of one’s social universe’:

[E]mployment status defined the identities, economic interests, social status and political loyalty of Yugoslav citizens. One’s place of work was the centre of one’s social universe. Social status, income guarantees, social benefits and political rights to participate in economic decisions and to be elected to legislative chambers for the economy varied according to the sector – social or individual – in which one was employed.^21^

Similar to other state socialist countries, factory-based welfare provision as well as the tying of everyday political, cultural and leisure activities to the factory premises, deepened workers’ attachment to their workplace. However, Yugoslav self-management offered an even more thorough identification of personal well-being with the business performance of the enterprise. Unlike the classical command economies of the Soviet type (where the decision-making in industry extended from the top planning bodies of the state to the factory shop floors), the Yugoslav system broke the command chain at the level of the company. Elected management was introduced which ostensibly ensured the freedom of autonomous business decision-making in the market. The professional management and the worker councils were thus in a position to build a base of support among their worker constituencies and frame popular grievances as a struggle for more business freedoms against the bureaucratic centralism of the party-state.^22^ In many successful factories, the directors ruled in a strong-handed manner, and although combative towards the self-management bodies, they nonetheless enjoyed great popularity on the shop floor.

The workers’ incentive to rely on the managerial elites to further their interests, rather than focusing on activity inside the self-management organs or the party-state, was of a practical and ideological nature. Despite perennial institutional reorganizations of self-management (the latest and the most ambitious approach being the 1976 Associated Labour Act)^23^ Yugoslav workers rarely had the opportunity to voice their opinions beyond the factory gate. Until the twilight years of the Yugoslav state in the late 1980s, the dominant understanding of workers’ self-management remained that of the enterprise’s right to decide over the usage of its total income and not that of workers’ political power and meaningful control of macroeconomic processes. A large percentage of the factory income hinged on a successful performance on the market where it competed with other producers, thus fostering collective identity at the company level and leaving manual workers dependent on experts.^24^

The pronounced discrepancies between the business successes of single enterprises, economic sectors and geographic regions emphasized the importance of local allegiance and discouraged the establishment of all-embracing working class fora in the sphere of self-management. The wages of workers with the same occupational positions in different enterprises located in the same city could vary greatly, while the divergence between the levels of economic development between the republics grew over time in spite of the professed aim of overcoming inherited regional inequalities.^25^ The fact that a great number of social services the Yugoslav state provided to its citizens were connected to the single enterprises made the inequalities between different sections of the working class even more pronounced.

In our research, we find that workers usually identified themselves and other workers primarily in relation to their enterprise. For instance, the workers of an automotive manufacturer TAM (*Tovarna avtomobilov Maribor*) in Slovenia were colloquially called *tamovci*’ while the workforce of the rival Serbian truck producer FAP (*Fabrika automobile Priboj*) embraced the informal name *fapovci* with great pride. Yugoslav socialism thus created a peculiar version of workplace allegiance and micro corporatism.^26^ The concept of ‘social ownership’ in the self-managed industry implied that the capital goods installed in the factories belonged to the society as a whole. The right to manage social property ended however with retirement and there was no way of privately owning or inheriting collectively managed capital. Yet, many workers had a narrower understanding of the concept. They felt a small part of the factory was indeed ‘theirs’ and it was a common practice to favour the children of employed and retired workers and pensioners when hiring new workers.

The Yugoslav self-managing practice therefore had the potential to unify the factory behind management and inspire workers to identify themselves with the enterprise. However, the atomization of the working class at the national level was paralleled by growing polarization inside the factory. Workers of a single enterprise were far from a homogeneous group with an equal sense of attachment to the shop floor, the factory product, and the enterprise. A layer of the Yugoslav industrial workers achieved great social mobility under self-management and internalized the ruling ideology in the process. These were usually male, senior workers with a vocational education or rare and valued skills acquired during the years spent on the shop floor. Over time, most of these workers managed to settle down close to the factory and integrate into the local community with the help of decent wages and heavily subsidized socially owned housing. They attended after-work events and leisure activities organized by the factory. As a rule, they were also active in various self-management organs and the party. As such, they were representative of the possibility of new consciousness of working people, surpassing a ‘wage earning mentality’ focused on self-interest and short-term gain.^27^

However, at the other end of the spectrum, we can observe younger workers, newly arrived to the city, anxious to attain the comforts of socialist modernity but finding them to be ever more elusive, due in part to the closure in social mobility.^28^ Their prospects of settling in industrial communities as full-time wageworkers were shattered by the harsh realities of factory work and the inability to advance to better-paid positions. The older, unskilled workers, earning low wages and residing in distant, semi-rural settlements, were also partly cut-off from the advantages of urban life and the socialized economy. Many of them were female workers whose work in feminized sectors like the textile industry was frequently poorly paid,^29^ or migrant workers from the peripheries of Serbia and other Yugoslav republics. These layers of the industrial working class rarely participated in self-managing decision-making and the systematic favouring of older and more skilled male workers made them sceptical toward the concept of shared enterprise interests or the idea of common progress through socialism.

A person’s place of origin and place of residence played a decisive role in determining whether they internalized the official discourse of the model shop floor worker who participates equally with their colleagues in production and appropriation of surplus value. Access to socially owned housing was probably the single most important factor in conditioning well-being and social status.^30^ All workers were formally entitled to socially owned housing which was financed and distributed by the factories. However, only a quarter of Yugoslavia’s housing stock was in the social sector and of the available stock, workers were systematically discriminated against in its allocation in favour of white-collar and skilled workers.^31^ The self-management bodies elected housing commissions which were in charge of distributing apartments through waiting lists. However, the housing development budgets were chronically underfunded and plans were rarely fulfilled. As in other socialist states, shortages rendered urban housing a scarce commodity whose distribution was regarded with suspicion, often pitting workers against each other and stirring regular complaints.^32^

The working-class households who remained on the housing waiting lists had to spend a large share of their earnings subletting an apartment on the grey rental market or independently constructing a family home (which would usually be located in semi-rural neighbourhoods far from the amenities of urban life and public infrastructure).^33^ A detailed description of the life of a migrant worker, published in the periodical of one of Belgrade’s metal factories (*Industrija motora Rakovica*) offers insight into the living conditions of many blue-collar workers at the time.^34^ Milovan Čolaković arrived to Belgrade from a rural part of Kosovo. After years of subletting rooms, he managed to build a house of his own in a village on the outskirts of Belgrade. The house was not yet completed and different parts were added each year thanks to the physical labour of the extended family. Milovan, his wife and their two children occupied the first floor. The second floor was left for his mother and younger brother. Like most workers, the family could not afford a car and depended on sparse public transport between their village and workplace. Many foodstuffs for the household were obtained at subsided prices through the trade union.^35^ Although the article does not mention it, Milovan, like many of his colleagues, probably also raised domestic animals like pigs and chickens and grew fruit and vegetables from his own garden.^36^

These workers with an ‘unresolved housing status’ often blended with another marginalized group known as ‘peasant workers’. The term referred to those blue-collar workers who continued to reside in villages and tended to neglect their shop floor responsibilities in favour of subsistence agricultural labour with their family and neighbours.^37^ The party-state and factory management tended to tolerate occasional absences and lower work efforts of the peasant workers as subsistence farming lowered wages and eased the pressure on the scarce goods and services of the urban settlements.^38^ Even those workers who managed to settle down in industrial neighbourhoods, and saw the factory as the centre of their life activities were often tempted to exchange their place behind the machine for deskwork by taking active part in self-managing bodies and becoming full-time functionaries. Moonlighting was also widespread and many industrial workers with a rural background chose to leave the factory as soon as they managed to find a more lucrative and higher paid job in the informal or service sector in the city.^39^

Other workers held a factory job and engaged in moonlighting to make ends meet. By the 1980s, the factory pay-check was frequently no longer a sufficient income. For example a clerk in ‘21 maj’ Rakovica, Danka Hadžimurtezić, stressed that although her young family lived with her husband’s father in Sremčica (a village close to Belgrade’s southern suburbs) and so enjoyed a cheap rent, the costs of repaying credit, electricity, fuel and childcare were significant and if her husband did not work privately in addition to his job in the social sector, ‘the situation would be very difficult’ for the family.^40^ A blue-collar member of the Serbian League of Communists Central Committee commented on moonlighting in 1986:

A large percentage of the working class has been living on the edge of poverty for quite some time now. The wage they receive at work does not enable them a decent living. They are unable to maintain their personal belongings and service their household appliances, not to mention buy new ones. Many are considering additional or alternative jobs. Our best craft workers are forced to work additional jobs in order to keep their families fed. If this process continues, there is a danger that these supplementary jobs might become the main ones and the factory work be reduced to a side activity.^41^

The theoretical prescriptions of global labour history, *inter alia*, emphasize the need to shift the focus away from the nation state as the primary unit of analysis when examining at the industrial workforce.^42^ This is particularly pertinent in terms of exploring labour and long-term patterns of migration across the Balkan peninsula.^43^ Significant number of Yugoslav blue-collar workers took advantage of the open-border policy to work in Western Europe as a ‘transnational Yugoslav working class’.^44^ As a consequence, since the late 1960s, industry was faced with the departure of many skilled manual workers who had left for better paid jobs in western Europe. This was not a unidirectional migration flow however. Workers would often travel back and forth, taking prolonged sick leave and using their holidays to go abroad and seek employment, comparing advantages of leaving their workplace back home. Others undertook regular seasonal jobs at home and abroad in order to boost their income or save for larger purchases like a house or household goods. As the economic crisis became more severe in Yugoslavia during the 1980s, some workers used the established migration channels in order to start petty smuggling rackets of consumer goods which were sold informally in factories.^45^

Despite great pushes toward industrialization and urbanization facilitated by socialist policies, a summary of the composition of the industrial working class in interwar Yugoslavia made by Marko Miljković in his research, could still be cautiously applied to significant segments of the working class in late socialism:

The statement might be hazarded that no class identity actually existed, except in some ‘pockets’ of relatively modern industrial facilities […] Low-skilled and poorly educated, this ‘industrial peasantry’ seems to be a highly diverse group of people in the beginning of the process of becoming urban industrial workforce, possibly only unified by a general resentment of factory life and by their need to find additional source of income, whether working in agriculture or in some other extra jobs during or outside of regular working hours, possibly even resorting to petty criminal activities.^46^

The failure of researchers dealing with Yugoslavia’s demise to recognize and bring to light the structural inequalities, persisting ambivalences and subdued dissatisfaction within the working class results in accounts of sudden breaks and ruptures which stress the power of elites to easily manipulate their constituents. Scholars have tended to conceive of the working class as either inherently nationalist and populist or alternatively, as having been seduced or manipulated by demagogic elites.^47^ However, by accounting for the ambivalences and seemingly contradictory trends in socialist labour inside the factory over the longue durée, the shifts and ruptures of late socialism appear more coherent. In the following section, we sketch some of the main methodological considerations, which might help researchers to detect these subtle processes when working with the sources at hand – namely archival holdings, print media and oral history.

## Approaching the sources: Archival holdings

Archival holdings relating to socialist enterprises in Yugoslavia tend to be inconsistent in scope. In principle, enterprises were expected to deliver documentation to their local historical archive. In practice, this was not usually carried through and in the postsocialist era it has been further complicated as factories have all undergone changes in ownership (from self-managing social ownership to state ownership and subsequent privatization processes which more often than not have resulted in firms being stripped for assets). In cases where a factory was privatized, the archival holdings are often swiftly disposed of by the new owners. Occasionally, the archives still remain within the factory.

**Figure 1.**
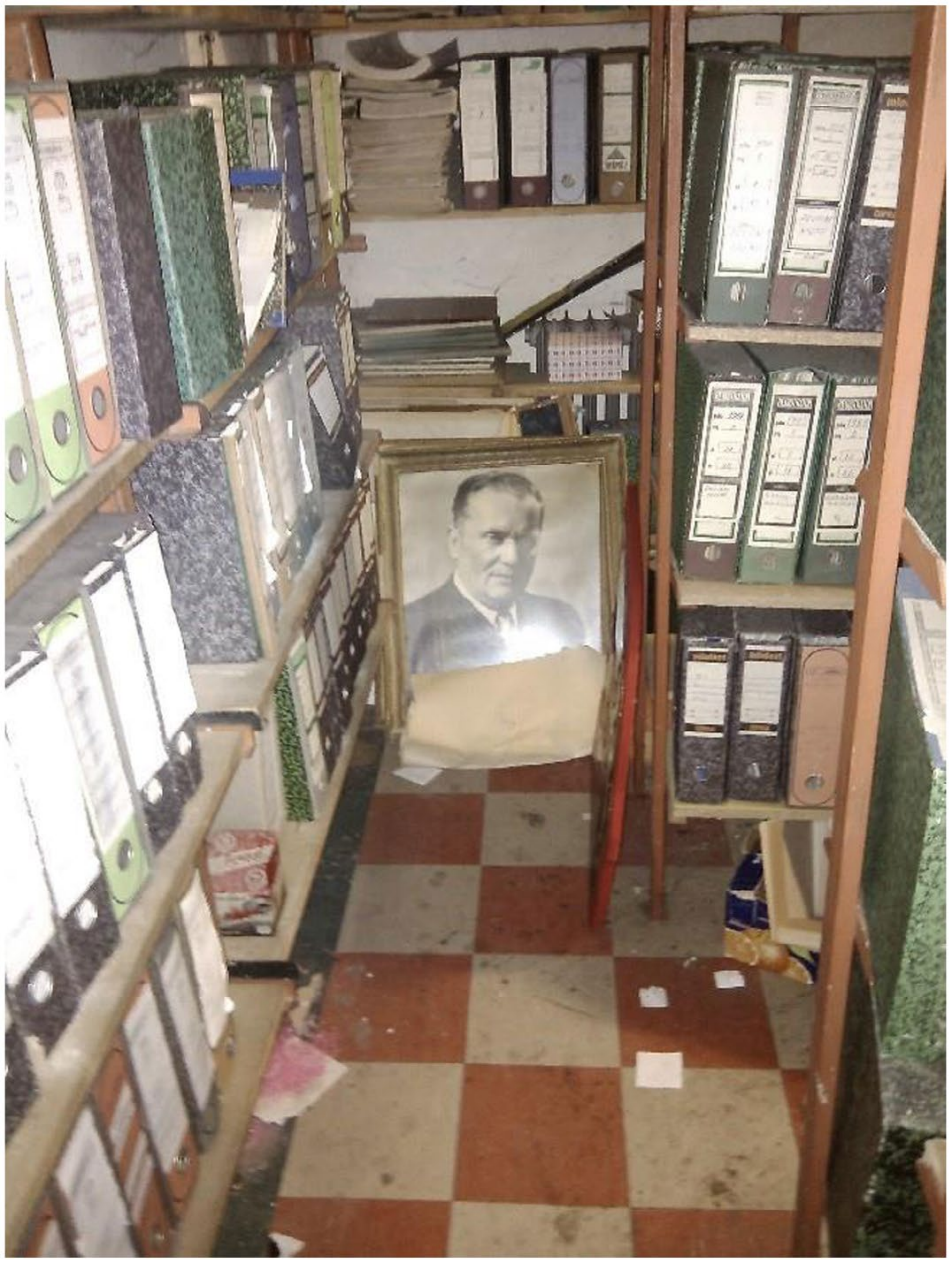
FAP archive, Priboj, April 2015.

Source: Photo by Rory Archer.

When the archives are still located within the factory, access is problematic and highly personalized (i.e. dependent on making the right connections to ensure access). We were fortunate to be able to access *in situ* archives in one case study location, Priboj in south-west Serbia (Sandžak). The ailing heavy vehicle manufacturer is owned by the Serbian state and in early 2016 is still awaiting privatization after numerous failed attempts to find a buyer and sporadic strikes. Through formal requests from our institution, the fostering of personal connections and good luck, one-time access was secured and we were able to access the factory archives in 2015 (Figure 1). Inside the archives however, we encountered numerous shortcomings. Much of the documentation was incomplete, disorganized, and usually tended to be biased towards management. For example, when exploring the institutions of self-management like workers’ council meetings, usually only final decisions tend to be recorded. Records of the debates, discussions and consultation processes with workers which led to such decisions are largely absent in the archives. It is these discussions that provide a more nuanced impression of factory dynamics and account for different interest groups, perspectives and motivations.

One way to mitigate the elite centred bias is to focus on the factory organizational units where actual production took place. In the Yugoslav context, this is made easier by the division of all workplaces after the 1976 Law on Associated Labour into atomized units called ‘Basic Organisations of Associated Labour’ (BOAL).^48^ A useful approach when exploring working-class concerns in the factory is to seek documentation related to the BOALs where production took place – e.g. the assembly line of a vehicle manufacturer rather than the accounts department or the general director (the latter documents often being prioritized in factory archives). BOALs from within the same factory were at times in antagonistic relations as a consequence of their diverse interests relating to the production process. Indeed, mapping out the differing interests and claims between BOALS can help illuminate the divisions and competing concerns within one factory (as well as how these relate to the wider community, indeed right up to the level of the state).

In addition to the factory-related documents, alternative and complementary sources include local municipal party and trade union records. With large enterprises at the centre of economic and political life in communities such documents can inform upon the relation between the enterprise and broader community. Similar to factory archives, such documentation can be unpredictable and incomplete. The municipal archives for the League of Communists in Priboj for example, were allegedly destroyed while en route to the local historical archives in Užice.^49^ For the purpose of reconstructing relations in the workplace and informing on everyday life in working-class communities, these documents can appear rather dry and concerned with hierarchical power structures. The patchy holdings of factory archives and local authorities can thus be further verified, contextualized and contrasted with oral history accounts, print media and factory periodicals.^50^

## Print media

The reflexive nature of Yugoslav socialism was reflected in a fairly lively press and although certain themes and registers of reporting were discouraged or expressly forbidden, print media remains a fruitful site for historical research of the socialist era factory. Research by Brigitte Le Normand indicates that local newspapers were a site for the discussion of workers’ housing issues in terms of the politically acceptable discourse of (in)equality at least form the early 1960s.^51^ By the 1970s social issues received increased public scrutiny in the press.^52^ Newspapers followed scandals and concerns in the workplace and beyond and so kept:

[…] their readers informed on which restaurants water their wine, what factories are wasting foreign currencies by buying too many foreign patents and how the communes are pressuring successful firms to merge with unsuccessful ones. Descriptions of embezzlement and corrupt directors running their worker’s councils are a standard feature in the campaign to strengthen workers’ self management.^53^

Press reports contain a wealth of empirical data including caustic editorials, human interest stories and reports on how the ordinary (wo)man viewed and were affected by various phenomena inside and beyond the factory perimeter.^54^ Yet perhaps even more valuable sources are those publications produced by, for and within the factory – workplace periodicals. These heterogeneous publications are the ‘bread and butter’ of much research of labour in Yugoslavia.^55^

### Workplace periodicals

**Figure 2.**
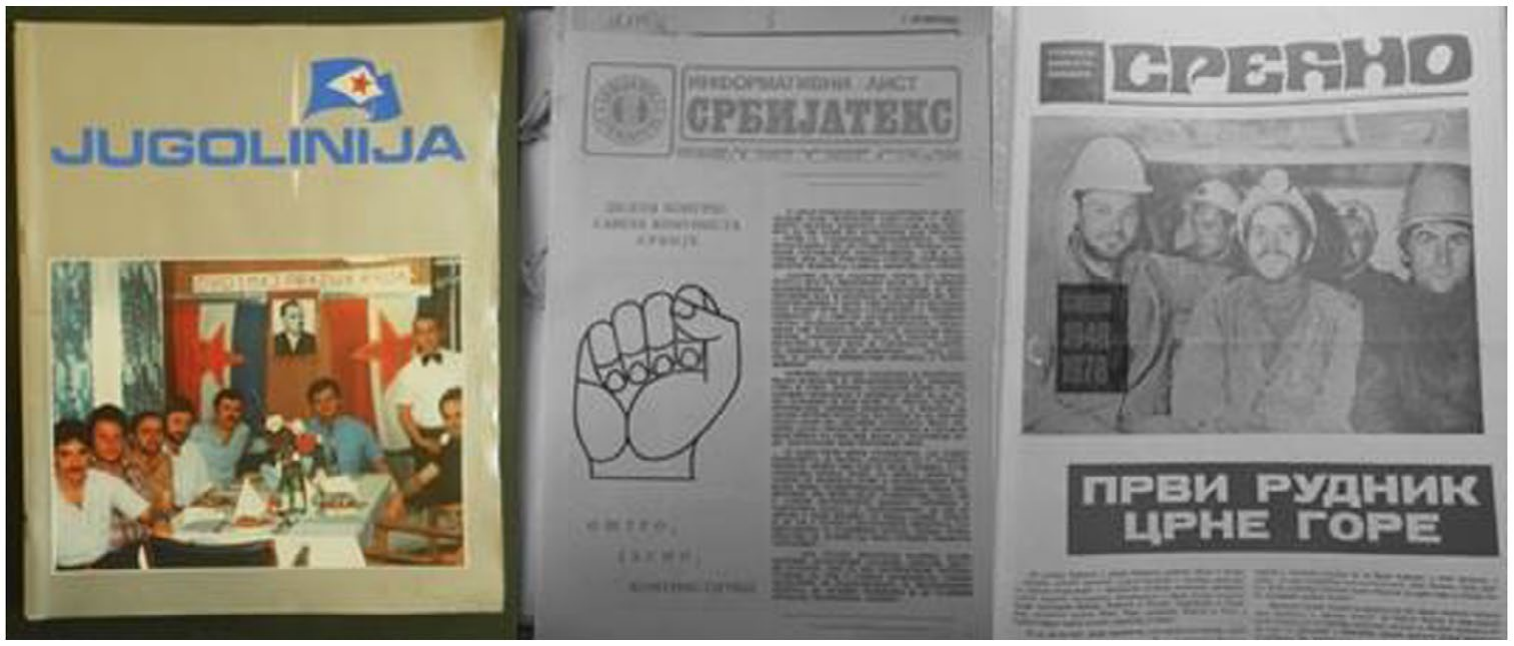
Front pages of workplace periodicals (Jugolinija, Rijeka, 1 May 1988; Srbijatekst, Belgrade, June 1986; Srećno, Nikšić, August–September 1978).

Yugoslav social sector workplaces published various documents to inform workers about their place of employment. Some of these were strictly internal documents or bulletins but by the 1970s larger workplaces published periodicals in a magazine or newspaper format (Figure 2). Many of the workplace periodicals emerging in this period referred explicitly to the 1976 Law on Associated Labour, Article 546 of which stated that basic organisations of associated labour (BOALs) were obliged to provide regular, timely, truthful content to workers in an accessible manner.^56^ The restructuring and decentralization of the Yugoslav economic system with this law provided an ideological impetus to regularly publish a periodical as a means of communication between the various BOALs, League of Communists, trade union organizations and other institutions of self-management associated with an enterprise.^57^

While one might expect these periodicals to exclusively represent management and craven party views they do nevertheless inform upon the concerns of workers and provide a range of viewpoints and critique, not only in and between the often unruly BOALs but also the wider community and state. Trade union periodical *Beogradski radnik* printed an article in 1983 about factory periodicals under the title ‘Factory periodicals: for the workers or directors? Something in between’.^58^ In addition to questioning whose interests the periodicals advocated, the issue of censorship was raised. *Beogradski radnik* investigated the recently published periodical of food manufacturing company Soko Štark which featured a report on a meeting in which the work of a director was sharply criticised. The article was censored with a black marker. While this was described as clear evidence of the director’s power, the director’s influence on the periodical was not without limits – in the following edition a Soko Štark worker’s letter of complaint about censorship was published: ‘What are you doing, editor?’^59^ Many publications explicitly stated their intention not to function as a ‘top-down mouthpiece’ but as a site of mediation between employees of various backgrounds and the atomized BOALs (indicative of the normative elements of the Law on Associated Labour which envisaged greater worker participation in decision-making at many different levels).^60^ Indeed a Serbian law on public information forbid that editorial work be undertaken by management.^61^

### Cartoons and informal content in print media

As well as including various procedural and administrative content from management and technocratic bodies workplace periodicals often reported on discussions and controversies inside the company and provided space for workers to contribute through writing texts as well as contributing jokes, aphorisms and cartoons. We conceive of these less formal contributions as a space where the articulation of worker dissatisfaction and frustration was particularly pronounced. Being mindful of the particular ideological and market constraints placed on the editorial boards of print media, a careful analysis can nonetheless inform upon some of the contentious issues of concern to workers. One can observe the interaction or convergence of institutionalized and informal practices in the workplace. Through wry humour, aphorisms and cartoons, one can detect a multiplicity of ambiguous (sometimes even openly antagonistic) attitudes towards management and technocratic elites as well as towards fellow workers and dominant practices of work and non-work. Such visual critique often went far beyond the bounds of what would be appropriate for the textual content. Yet, in the workplaces we have explored, the cartoons and informal content do not appear to have caused major controversy.

Just as one might consider cartoons as a means of asserting the agency of the weak, we should also not discount the possibility that cartoons and informal content in workplaces represented a form of depoliticization by identifying topical issues and providing a valve for their articulation in a way which was unthreatening to those wielding social power. Rather than considering cartoons as a conduit to bring new issues to public light, it should be acknowledged is that ‘the idea contained in a political cartoon must not only be easily understood but even be already widely established *before* the cartoonist uses it’.^62^ Clearly, the ideas expressed in the cartoons featured in workplace periodicals held currency before their publication. They took their lead from a popular repertoire of ideas and representations already in public circulation. Nevertheless, the cartoons in question were clearly of a political nature and demonstrated politicized content. The issues broached (like housing difficulties, worker discontent and conflict in the workplace) were all seen as political controversies bound up in the interlocking social, economic and political crises of the 1980s. The semi-public discussions taking place in workplaces across Yugoslavia through public meetings and workplace periodicals addressed not only issues of concern for the particular enterprise but for society as a whole and this is reflected in the cartoons which demonstrate a broader social commitment.

**Figure 3.**
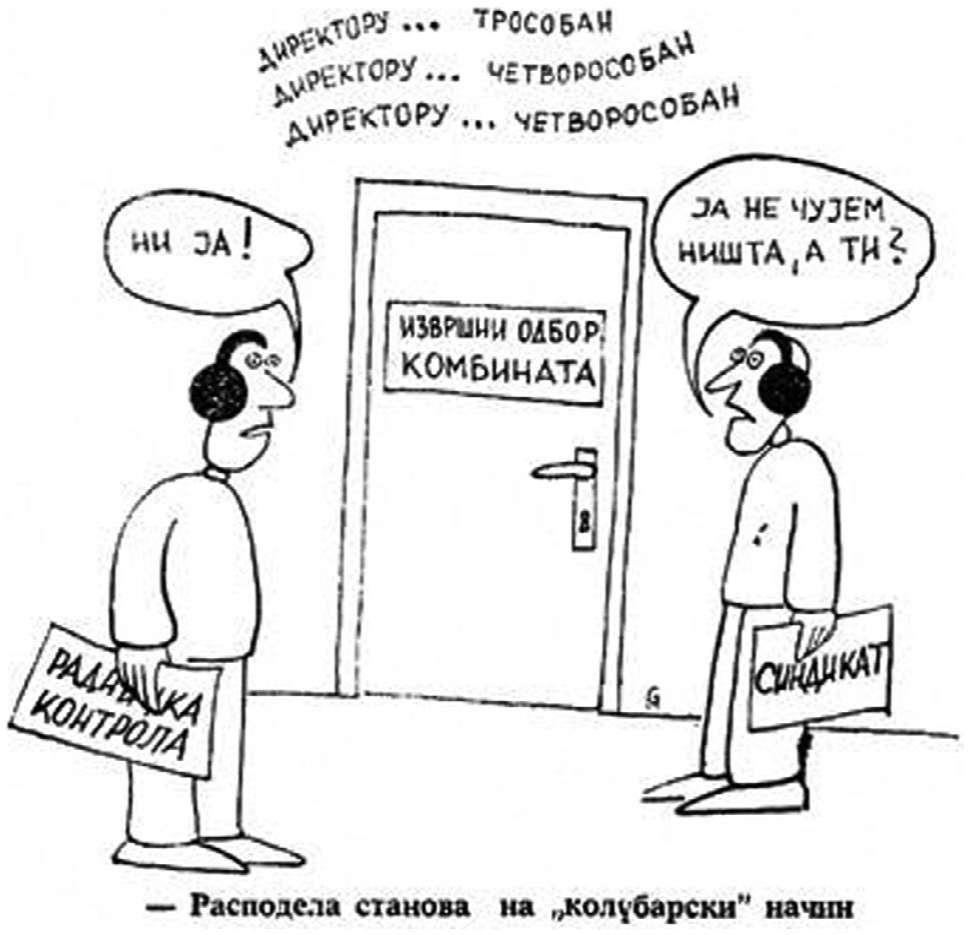
Cartoon from workplace periodical *Kobubara* (August 1978) ‘The distribution of flats the “Kolubara” way’.

Source: Živanović, “Kako je Milan Gambelić osetio moć karikature”.

It is important to keep in mind the nature of the socialist pluralism (or ‘laissez-faire socialism’^63^) espoused by the Yugoslav system. A certain amount of open discussion, criticism [*kritika i samokritika*] and even open verbal conflict was seen as desirable, provided it was in the spirit of Yugoslav socialism. Because dynamics like the dichotomy between blue and white-collar workers resonated according to official ideology, playing with such divisions could be tolerated, indeed encouraged amongst various actors.^64^ The cartoon above for example, sketched in 1978 by young Kolubara engineer Milan ‘Miša’ Gambelić, illustrates the ‘distribution of flats the Kolubara way’ at the Serbian lignite field (Figure 3). Behind the door to the general director’s office one can hear ‘for the director, a three-bedroom flat, for the other director, a four-bedroom…’ Management was corruptly divvying up socially owned property with the acquiescence of the trade union representative and work controller who were pretending not to hear anything. According to the accompanying article the cartoon prompted a review of flat distribution processes in Kolubara. Gambelić was not sanctioned; in fact his cartoon was allegedly accepted as ‘well intended criticism’.^65^

In addition to a more reflexive ideological matrix, it is also possible that the Yugoslav authorities simply regarded images like cartoons as less threatening than printed text. The level of critique and cynicism displayed is arguably far more brazen than anything in the accompanying texts which usually frame issues in a moralistic socialist language register. In her study of visual memories of communism in Romania, Simina Bădică found that in the 1980s, the Ceaușescu regime was extremely fearful of the printed word. Typewriters had to be registered with the police annually, a process she likens to gun control regulations. At the same time, the authorities not only tolerated photography but even endorsed amateur photography as a pastime and cameras did not have to be registered.^66^ Perhaps a similar (albeit less extreme) dynamic can be observed in Yugoslavia whereby images, in this case cartoons, were viewed with less caution by authorities and thus could risk being more explicit and biting than their associated texts.

## Oral history

Many of the most potent themes and representations found in cartoons and other informal content are mirrored in oral history research about the factory. There are notable similarities between the attitudes expressed by working class oral history narrators and the informal content of workplace periodicals (and sometimes notable gaps and silences amongst both). In both workplace cartoons and oral history accounts issues like divisions between workers and management, laziness, ‘negative phenomena’ like theft, corruption and absenteeism are frequently cited and drawn. By the same token however, certain sensitive issues like ethno-nationalism and sexuality are skirted over (despite other sources suggesting these were also very topical issues). ^67^

Yugoslav sociological and historical enquiry has tended towards quantitative and empiricist forms.^68^ Thus, very little research exists which explores the lives of workers in a qualitative sense. Oral history is well poised to capture previously neglected facets of social life in Yugoslavia, particularly given the liminal context observed by Ljubica Spaskovska whereby ‘the Yugoslav time is historical, while the (post)Yugoslav space and many people who inhabited that time and space are still in existence’.^69^ Yet this liminality may be a source of discomfort for positivist historians. Although historiography of late socialist Yugoslavia and its dissolution has relied on interviews with political and intellectual elites and community leaders,^70^ oral history has remained comparatively marginal (with the notable exception of Magid’s oral history focusing on the legitimacy of the Yugoslav system).^71^ A frequent criticism of oral history has been the divergence between oral sources and documentary evidence (in which documentary evidence is prioritised). The typical argument maintains that oral history ‘cannot rank with an authentic diary, with a contemporary stock report, or with an eyewitness account transcribed on the day of the event’.^72^

The researcher might productively engage in a project that employs oral history as the primary method and source (indeed a plethora of studies relying on oral accounts of labour and everyday life may serve as inspiration for such an approach).^73^ The methodological choice we have made in our research, however, is to combine oral history with other sources and methods. When working with an array of sources, oral history research can help to fill in blanks and omissions in documents as well as leading the researcher to other documents and providing textured detail about narrators’ subjectivities. Furthermore, oral history is particularly well poised to gain insight about individuals and groups who tend to be marginalized from the historical record. It ‘allows us to get at the valuable knowledge and rich life experience of marginalized persons and groups that would otherwise remain untapped, and, specifically, offers a way of accessing subjugated voices’.^74^ Alastair Thomson writes that,

[t]he lived experience of working class, women’s or black history was undocumented or ill-recorded and oral history was an essential source for the ‘history from below’ fostered by politically-committed social historians in Britain and around the world from the 1960s onwards.^75^

In factory case studies in socialist contexts like Yugoslavia, marginalized groups might include low-wage workers (in production and out of it), in particular female workers. Oral history can offer insight into phenomena which were not considered relevant or appropriate and thus are absent or marginal in print media and in the archives (for example issues like sexuality, childcare, the gendered division of labour and ethnonational divisions). Oral history also enables the researcher to tease out workers’ subjectivities, understood by Alessandro Portelli as ‘the study of the cultural forms and processes by which individuals express their sense of themselves in history’.^76^ For example, sisters Mirjana and Gordana spoke about working in a Belgrade wood processing collective in the 1970s and 1980s. They recalled sexual harassment by managers and the trials of single parenthood in the self-managing workplace. Mirjana’s experience of joining and subsequently leaving the League of Communists reveal how some of the permutations of class and gender were experienced. As a cleaner and courier, she was encouraged to join the party in her workplace to bolster the numbers of rank and file workers. By 1985, however, Mirjana was extremely embittered by management who she believed were defrauding the collective and lying to the workers. Furthermore, she considered that her role as a party member was more of a burden than a privilege.

As I was in the Party I was supposed to be a good example to other workers, not allowed to make any mistake. Because of [going for] a coffee for example, I faced a disciplinary procedure, because I drank a coffee! … I said to the director then, do you know what? You drink coffee as well during the working hours! I knew this because I brought them the coffee.^77^

Membership in the League of Communists could expose a worker to increased scrutiny but still not necessarily guarantee meaningful privileges. As a working-class woman, Mirjana felt that she could not access the privilege of membership like her senior colleagues (sitting down for a coffee spontaneously at work) but at the same time membership inhibited her partaking in the same activities as her working-class colleagues who were not party members and so did not have to act as model workers.

Of course, a number of disadvantages and pitfalls are evident in oral history research of the socialist factory. One significant difficulty is access to oral history narrators in large urban settings. Many factories closed more than two decades ago and substantial demographic transformations have occurred throughout former Yugoslavia rendering it difficult to make contact with potential narrators. Access to narrators is far easier to achieve in small ‘one factory’ towns like Priboj but becomes difficult not only in large urban centres like Belgrade but even in mid-sized (formerly) industrial towns like Zrenjanin. The oral history approach thus demands that the researcher has ethnographic competence, sufficient time and a social network through which to make contact with potential narrators.

When engaging in oral history research a further challenge is the spectre of postcommunist nostalgia.^78^ The socio-economic difficulties and factory closures since 1990 as well as the Yugoslav wars of the 1990s may prompt narrators to recall the socialist past in overly positive terms while glossing over the negative features of everyday life in the late socialist factory. We noticed this in Priboj whereby individuals regularly made outlandish claims regarding the prosperity of the municipality within socialist Yugoslavia which we knew to be untrue. Such a phenomenon is probably widespread across postsocialist eastern Europe. As David Kideckel observes in his ethnographic study of miners of the Jiu Valley in Romania, despite the brutal experience of late socialism under Ceaușescu, (former) workers ‘readily downplay the state’s past coercion to look favourably on the security and enabling features of socialism’.^79^ Given the less coercive context of socialist Yugoslavia such claims are probably even more exaggerated.

There are two approaches we have used to counteract such exaggerations. The first is to triangulate oral accounts with other sources. Newspapers, workplace publications and archival documents can help contextualize, correct and counteract the excesses of nostalgia (and the unreliability of memory more generally). A second approach is the particular strategy of conducting oral history. Keeping in mind Trevor Lummis’s distinction between ‘memory’ and ‘recall’, the focus on a detailed life history (in terms of education, access to employment, housing, health care, biographical progression, etc.) is used as a methodological tool. It seeks to generate ‘responses to detailed interviewing which prompts dormant ‘memories’ that are less likely to be integrated into the individual’s present value structure’ (as opposed to more polished structures which suggest the narrative has been retold or thought about).^80^ For example, although narrators Mirjana and her sister Gordana are extremely critical of neoliberalizing Serbia and believe that opportunities for a ‘normal life’ for their children – to get a job and raise a family with dignity – have been massively curtailed, their own personal accounts of work and everyday life in the 1980s were not recalled through a nostalgic lens. When expressing their own lived experience of labour, (not) accessing housing and living in Belgrade’s peripheries in the 1980s their personal accounts are often rather critical. Our experience in the field thus far suggests that a focus on one’s lived experience can temper some of the unrealistically positive portrayals of the recent Yugoslav past and result in more nuanced accounts of labour and everyday life.

## Conclusion

This article has argued that the socialist era factory and its surrounding community of workers, former workers and their families remains an insightful location for research, not only in terms of labour history, but regarding the rise and fall of socialism in broader terms and particularly in relation to working-class subjectivities. By focusing on the industrial plants and working-class communities, one can gain a better insight into the heterogeneous ways that macro-level processes and events were experienced at the micro-level. Despite advances in the exploration of labour in state socialism, the micro-level remains understudied, particularly in the Balkans where the study of labour has paled in comparison to its more sophisticated treatment in central and eastern Europe.

We have sought to convey the complexity of the Yugoslav working class, the object of great veneration during the existence of state socialism which today remains curiously undertheorized in scholarly literature treating late Yugoslav socialism and state dissolution. We have also called for avoiding essentialized and homogenized representations of the working class although acknowledging that the official ideology of the party-state did have (and probably still has) an impact on the ways the working class perceived itself and the modes in which individual workers engaged with other social actors. As Brunnbauer writes in relation to communist Bulgaria, official knowledge is ‘also essential for understanding social relations under socialism’ in spite of the unreliable nature of information which circulated in the public domain.^81^

Through our experiences of conducting fieldwork in the form of factory case studies in a (post-) Yugoslav context, we have outlined some considerations with regard to approaching the sources. In a fragmented setting like the former Yugoslav states and their postsocialist neighbours, researchers need to be flexible in terms of access to sources and their analysis. In certain communities surrounding (former) factories, the absence of printed and archival sources demands an engagement with oral history methodology. In other locations, particularly larger towns and cities, an oral history approach is more challenging but accessing documents is perhaps more feasible. In any case, the researcher is likely to rely on a mix of methods.

There are clearly specificities to Yugoslavia which are unique in the socialist world and the limits to which our observations can be generalized for other socialist states in central and eastern Europe needs to be acknowledged. In Yugoslavia from the 1950s onwards, self-managing socialism (and market socialism from the 1960s) fostered a relatively reflexive style of public discussion which encouraged the development of higher levels of personal autonomy and liberalism in comparison to other states in central and eastern Europe (in particular the austere variants of socialism as practiced in Yugoslavia’s neighbours Albania, Romania and Bulgaria). Nevertheless, certain dynamics of the Yugoslav factory and society – such as the fragmentation of the working class, high fluctuation of the workforce, the informal economy, low work discipline, bureaucratization and parochialism – remain common to Warsaw Pact members and we believe that there are sufficient commonalties to facilitate some guarded generalisations. A productive next step would be to further compare and contrast the Yugoslav factory with industry in other socialist contexts to test some of these observations.

## Notes on contributors

***Rory Archer*** works as a researcher at the Centre for Southeast European Studies, University of Graz, on the project ‘Between class and nation. Working class communities in 1980s Serbia and Montenegro’ (funded by the Austrian Science Fund – FWF). He holds a PhD in History from the University of Graz. His research interests include Labour History and Everyday History of Socialism. His most recent publication is *Social Inequalities and Discontent in Yugoslav Socialism* (coedited with Igor Duda and Paul Stubbs, 2016).

***Goran Musić*** is a researcher at the University of Graz’s Centre for Southeast European Studies working on the Austrian Science Fund (FWF) financed project ‘Between class and nation: Working class communities in 1980s Serbia and Montenegro’. He holds a PhD in History from the European University Institute (EUI), Florence. His fields of interest are Global Labour History, Comparative History of Workers under State Socialism and Everyday History of Socialist Yugoslavia. Recent publications include ‘“They came as workers and left as Serbs”: the role of Rakovica’s blue-collar workers in Serbian social mobilisations of the late 1980s’ in *Social Inequalities and Discontent in Yugoslav Socialism* (eds. Rory Archer et al., 2016).

## Disclosure statement

No potential conflict of interest was reported by the authors.

## Funding

This work was supported by the Austrian Science Fund (FWF) as part of the research project ‘Between class and nation: Working class communities in 1980s Serbia and Montenegro’ [FWF: P27008].

## Notes

1. See Kotkin, *Magnetic Mountain,* 203.

2. Rutar, “Towards a South East European History of Labour.”

3. On this project see: *YuLabour*, a blog following the research activities of the project ‘Between class and nation: Working class communities in 1980s Serbia and Montenegro’ https://yulabour.wordpress.com/

4. On the relationship between the state and self-management practice see Upchurch et al., ‘Spectrum, Trajectory’.

5. Brunnbauer, Raeva, and Visar, “Workers, Steel Factories and Communism,” 8.

6. See Katzenstein. *Corporatism and Change*; Blom-Hansen, ‘Still Corporatism in Scandinavia?’

7. Beyrau, ‘Das sowjetische Modell’, 64.

8. Ost, ‘Class after Communism: Introduction to the Special Issue’.

9. Pittaway, ‘Introduction: Workers and Socialist States’, 1.

10. Heumos, ‘Arbeitermacht im Staatsozialismus’, 51.

11. For an informative overview of the state in labour history of former socialist states and their particular traditions, see Heumos, ‘Workers under Communist Rule’.

12. In the case of the Soviet Union, scholars tackled labour and everyday life from earlier stages, see for example Kotkin, ‘One Hand Clapping’; Filtzer et al., *A Dream Deferred*.

13. See for example: Kenney, *Rebuilding Poland*; Pittaway, *The Workers State*; Heumos, ‘State Socialism, Egalitarianism, Collectivism’.

14. Yurchak, *Everything was Forever*, 4.

15. Jarausch, ‘Care and Coercion’.

16. Bren and Neuburger, ‘Introduction’ in *Communism Unwrapped*, 12.

17. See Bartha, *Alienating Labour.*

18. Heumos, ‘Workers under Comunist Rule’, 102–6.

19. Gilbert, ‘The past in parenthesis’, 14–8.

20. Musić, ‘They Came as Workers and Left as Serbs’, 132–3.

21. Woodward, ‘The Political Economy of Ethno-Nationalism’, 76.

22. Turkish-Comisso, *Workers’ Control under Plan and Market*.

23. Jović, *Yugoslavia: A State that Withered Away*.

24. Prout, *Market Socialism in Yugoslavia*, 50–8.

25. Pleština, *Regional Development in Communist Yugoslavia*.

26. Grdešić, ‘Exceptionalism and Its Limits’.

27. Turkish-Comisso, *Workers’ Control under Plan and Market,* 179.

28. Lazić, *U susret zatvorenom društvu?*; Dević, ‘What Nationalism has Buried’.

29. Bonfiglioli, ‘Gendered Citizenship in the Global European Periphery’.

30. Archer, ‘Paid for by the Workers, Occupied by the Bureaucrats’, 62–6.

31. Ibid.

32. For example, see: Harris, *Communism on Tomorrow Street*; Pelikánová, ‘Housing as a Norm and as an Everyday Life Strategy’.

33. See Tanić, *Beogradska predgrađa u prigradska naselja*; Saveljić, *Beogradska favela.*

34. 
^’^Ruke za dva takta’, 6.

35. Ibid., 6.

36. On the ‘rurbanisation’ of Yugoslav towns see Simić, *The Peasant Urbanites*.

37. Rural origins were deemed partly responsible for high turnover of the labour force as farmers left factories to work in agriculture during harvest season. Brunnbauer describes this phenomenon in Bulgaria: Brunnbauer, ‘The Town of the Youth’, 105.

38. Schierup, ‘Quasi-Proletarians and a Patriarchal Bureaucracy’.

39. Musić, *The Self*-*Managing Factory after Tito*, 126.

40. Jugović, ’Žena i njeno mesto u životu i radu ovog kolektiva’.

41. Ž.D., “Sa sednice CK SK Srbije: Opredeljenja sprovoditi u praksi”.

42. See: van der Linden, ‘Introduction’; Rutar, ‘Towards a South East European History of Labour’.

43. Brunnbauer ‘Labour Emigration from the Yugoslav Area from the Late Nineteenth Century until the End of Socialism’; Halpern, ‘Yugoslav Migration and Employment in Western Europe’.

44. Le Normand, ‘“The *gastarbajteri* as a Transnational Yugoslav Working Class’.

45. For example, a light-hearted comment in the periodical of engine producer ‘21 maj’ in Rakovica in 1980 suggested that selling smuggled goods was a common side business for coffee makers and couriers who were selling ‘better goods than at the flea market’ and bringing the flavour of Ponta Rosso (a shopping area of Trieste) into the Belgrade workplace. T.P., ‘Smešno ćoše’.

46. Miljković, *Western Technology in a Socialist Factory*, 39.

47. In Serbia, for instance, there is a notable historiographical trend of connecting Milošević’s rise to power with the political support from the lower social strata, the blue-collar workers in particular, see Musić, ‘They Came as Workers and Left as Serbs’, 132–3.

48. The Basic Organisation of Associated Labour (BOAL) was the smallest unit of the enterprise which constituted a political and economic entity. Each of these smaller enterprise departments was equipped with its own set of self-management bodies and joined the larger collective voluntarily, based on a self-management contract and a delegation in the central workers’ council. The Yugoslav self-managing enterprise in late socialism was a ‘work organization’ – an association of BOALs enjoying full legal and political sovereignty. ‘BOAL’ is also abbreviated as OOUR from the Serbo-Croatian *Osnovna organizacija udruženog rada*.

49. Personal communication with a well-informed archivist.

50. Another potentially fruitful archival source might include the data-sets and other field data used by sociologists of labour working in Yugoslavia during the 1970s and 1980s (we are grateful to Reviewer 1 for stressing this). Numerous studies were undertaken by sociologists on labour and self-management bodies and in the absence of meaningful developments in Yugoslav labour history during socialism, research by scholars like Mladen Lazić, Vladimir Arzenšek and Veljko Rus remains extremely valuable for labour historians. Smaller research projects were commissioned by local factory authorities for internal use and are sometimes are found in the factory archives.

51. Le Normand, “The House that Socialism Built”; Le Normand, *Designing Tito’s Capital*, 152–3.

52. Robinson, *Tito's Maverick Media*, 122.

53. Ibid.*,* 122.

54. For example, Yugoslav women’s magazine Bazar regularly included features on workers. An article from March 1989 covered female workers in FAP Priboj noting their advancement in the factory over the last decades but also acknowledging the physical challenges of working on the shop floor. Milošević, ‘Žene osvajaju FAP’

55. As evidenced in discussions with colleagues at a workskop *The history of labour in Yugoslav late socialism* (Pula, 2 October 2015). See: https://yulabour.wordpress.com/2015/10/05/the-history-of-labour-in-yugoslav-late-socialism-pula-2-october-2015/

56. Tiosav Velimirović et. al., *Zakon o udruženom radu*, 344–6.

57. In Belgrade social sector enterprises 118 periodicals were regularly published. While 3 were published on a weekly basis and 19 twice monthly, the majority – 80 periodicals – were published monthly. In larger firms special teams of editors and journalists were employed. See Aćimović, ’Fabrička štampa: radnička ili direktorska?’, 28.

58. Aćimović, *Beogradski radnik*, 28.

59. Ibid., 28.

60. The first issue of the *Jadrolinija* ferry company periodical in 1976 mentions the importance of ‘strengthening the influence of workers’ in the publication’s content and informing workers as part of a ‘battle against all attempts of individual and group influence of technical-bureaucratic structures’ on the flow of information. It was also deemed necessary to fight against ‘censorship and the holding back of information under the guise of “business secrets”’. The expression ‘interpersonal informing’ was used to stress the ‘wish that the list not get a one-sided character by informing ‘from the top’… from the perspective of individual leaders to ‘those below’. *Jadrolinija: informativni list organizacije udruženog rada*, 1 (June 1976), 1; Similarly, the newly launched periodical of textile enterprise Srbijateks expressed a wish ‘not to only publish decisions or opinions but enable workers to freely and openly collaborate by giving their thoughts and opinions’. See Tri decenije rada i uspona“ 1–2.

61. Aćimović, *Beogradski radnik*, 28.

62. Kenny and Colgan, ‘Drawing blood’, 229. Emphasis in original, cited in Curticapean, ‘Walls Doors and Exciting Encounters’, 114.

63. Rusinow, *The Yugoslav Experiment*.

64. For example, NIN’s foreign affairs editor Miodrag Marović, discussion the Polish uprising in 1980, noted that conflict between the working class and its ‘vanguard’ (the party) was possible, even ‘inevitable under specific historical conditions’. ‘The Marxist classics and even Lenin himself warned that the working class might be forced to defend itself from the tyranny and power of its own bureaucracy during the period of building socialism’. Antić, ‘Yugoslav Paper notes class antagonism in Poland’.

65. Živanović, *‘*Kako je Milan Gambelić osetio moć karikature’.

66. Bădică, ‘Forbidden Images?’, 208.

67. Archer, *Housing, social inequalities and discontent*, 97–120.

68. Archer et al., ‘Bringing Class Back In’.

69. Spasovska, ‘The Yugoslav Chronotope’, 241.

70. Dragović-Soso, *Saviors of the Nation*; Jović, *Yugoslavia: A State that Withered Away*; Vladisavljević, *Serbia’s Antibureaucratic Revolution*.

71. Magid, *Private lives/public surfaces*.

72. Grele, ‘Movement without Aim’, 41.

73. For example, see Bodnar, ‘Power and Memory in Oral History’; Jambrešić Kirin and Blagaić, ‘The Ambivalence of Socialist Working Women’s Heritage’; Koleva, ‘My Life Has Mostly Been Spent Working’; Passerini, ‘Work Ideology and Consensus’.

74. Leavy, *Oral History*, 151.

75. Thomson, “Four Paradigm Transformations in Oral History,” 51.

76. Portelli, *The Death of Luigi Trastulli and Other Stories*, ix.

77. Interview with Mirjana and Gordana, Belgrade, April 2014.

78. Velikonja, ‘Lost in Transition’.

79. Kideckel, *Getting by in Postsocialist Romania*, 17–8.

80. Lummis, ‘Structure and Validity in Oral Evidence’, 274.

81. Brunnbauer, ‘The Town of the Youth’, 92.
